# Supplementary material for: Mechanisms governing the pioneering and redistribution capabilities of the non-classical pioneer PU.1
Source: Nat Commun. 2020 Jan 21;11:402. doi: 10.1038/s41467-019-13960-2 (PMC6972792; doi:10.1038/s41467-019-13960-2)
Supplement: Supplementary file 7 — Source data [file 41467_2019_13960_MOESM7_ESM.zip › Source_Data/Figure6/Figure6B_MotifScanOutput/ETS-specific/homerResults/motif32.similar.html]

motif32

## Information for motif32

A
G
T
C
T
A
C
G
A
T
C
G
A
T
G
C
G
C
A
T
T
A
G
C
A
G
C
T
A
G
C
T
A
G
C
T
G
A
T
C
T
A
G
C
A
T
C
G
A
T
C
G
  
Reverse Opposite:  

T
A
G
C
A
T
G
C
A
T
C
G
C
T
A
G
T
C
G
A
C
T
G
A
T
C
G
A
A
C
T
G
C
G
T
A
T
A
C
G
T
A
G
C
A
T
G
C
T
C
A
G
  

|  |  |
| --- | --- |
| p-value: | 1e-30 |
| log p-value: | -6.935e+01 |
| Information Content per bp: | 1.706 |
| Number of Target Sequences with motif | 158.0 |
| Percentage of Target Sequences with motif | 2.06% |
| Number of Background Sequences with motif | 236.4 |
| Percentage of Background Sequences with motif | 0.59% |
| Average Position of motif in Targets | 108.3 +/- 47.6bp |
| Average Position of motif in Background | 93.4 +/- 68.6bp |
| Strand Bias (log2 ratio + to - strand density) | 0.5 |
| Multiplicity (# of sites on avg that occur together) | 1.06 |
| Motif File: | file (matrix) reverse opposite |

### Similar de novo motifs found

|  |  |  |  |  |  |  |  |
| --- | --- | --- | --- | --- | --- | --- | --- |
| Rank | Match Score | Redundant Motif | P-value | log P-value | % of Targets | % of Background | Motif file |
| 1 | 0.942 | C G A T G A T C T A C G A T C G A T G C A G C T T G A C A C G T A G C T A G C T A G T C A G T C A C T G A C T G | 1e-15 | -35.814579 | 0.54% | 0.07% | motif file (matrix) |
